# Reply to the letter to the Editor: Advancing deep learning-based segmentation for multiple lung cancer lesions in real-world multicenter CT scans

**DOI:** 10.1186/s41747-025-00650-6

**Published:** 2025-11-05

**Authors:** Xavier Rafael-Palou, Ana Jimenez-Pastor, Luis Marti-Bonmati, Carlos F. Muñoz-Nuñez, Mario Laudazi, Angel Alberich-Bayarri

**Affiliations:** 1 Research and Frontiers of AI, Quibim, Valencia, Spain; 2 Radiology Department, La Fe University and Polytechnic Hospital, Valencia, Spain; 3 Biomedical Imaging Research Group (GIBI230) La Fe Health Research Institute, Valencia, Spain; 4 Diagnostic Imaging Unit, Department of Biomedicine and Prevention, University of Rome Tor Vergata, Rome, Italy

Dear Editor,

We thank Xiaowei Huang and Xian Gu for their letter and their interest in our publication “Advancing deep learning-based segmentation for multiple lung cancer lesions in real-world multicenter CT scans” [1]. The points raised emphasize important considerations for rigorous AI evaluation in oncology imaging. We appreciate the opportunity to address these points.

The authors emphasize the need for patient-level clinical utility analysis to enhance interpretability [2]. Some works like ours are primarily methodological, aiming to develop and evaluate a robust multi-instance lung cancer lesion segmentation approach on heterogeneous, real-world data. The focus was therefore on technical performance and reliability, with analyses and metrics aligned to demonstrate this objective. Accordingly, a full assessment of clinical utility, particularly in longitudinal scenarios, was left for future work. In this regard, we would like to clarify that each patient in our cohort contributed a single scan image. Therefore, patient-level analyses were explicitly computed, corresponding to image-level metrics, including Dice scores (Table 2) and free-response ROC curves to characterize detection sensitivity and precision across scans (Fig. 5b). Additionally, Bland–Altman and correlation analyses comparing predicted and reference lesion sizes provided an initial assessment of volumetric calibration—an essential foundation for clinically grounded evaluations (Fig. 5c, d).

We also recognize the importance of acquisition- and device-related parameters in multicenter studies. In line with this, key parameters were quantified across both the main and external cohorts, although some features (i.e., inspiratory level) were unavailable or incompletely documented. Their letter also underscores the need for protocol-balanced splits and stratified performance to ensure generalization. To address this, we employed a random, stratified partitioning strategy for both the independent train-test split and K-fold cross-validation, using data source and slice-thickness to ensure proportional acquisition conditions represented across sets, thereby mitigating spectrum bias. Hence, segmentation and detection performance were evaluated on the heterogeneous cohorts at both lesion and patient levels, and generalization was further supported by consistent results on a real-world external dataset. We agree that future stratified analyses by additional acquisition and device-related factors could offer a more granular view of model performance [3].

Regarding the potential risk of pseudo-replication from multiple lesions per patient, we paid particular attention to the non-independence introduced by patients harboring multiple lesions (whereas multiple scans per subject were not present in our cohort). First, we provided a transparent description of cohort characteristics, including lesion counts per scan (IQR between 1–4 lesions ≥ 10 mm across all data source partitions). Second, as previously mentioned, complementary performance metrics and curves analysis at both patient and lesion levels were conducted to provide a detailed evaluation of method performance. We acknowledge, however, the added value of future analyses accounting for clustering by data source, patient, or lesion multiplicity per patient, which could further enrich the interpretability and comparability of reported results [4].

On the topic of inclusion/exclusion transparency, we extensively reported cohort characteristics at the data-source level for both development and external cohorts. Scan-level exclusions were applied strictly on the basis of objective image quality criteria, namely corrupted DICOM files, incomplete lung coverage and severe motion artifacts. By restricting exclusions to these reproducible technical criteria, we sought to minimize selection bias. Nonetheless, we agree that a dedicated flow diagram and a comparison of included versus excluded scans across key covariates would further strengthen transparency. In this same comment, more details on missingness were required. Here, we clarify that our segmentation framework was designed to rely exclusively on imaging data, and clinical covariates were neither incorporated nor required for model development. Consequently, issues of systematic missingness in covariates did not arise, and statistical techniques for handling missing data (e.g., multiple imputation or inverse-probability weighting) were not applicable in this context [5].

The authors rightly emphasize the need for temporal validation in oncology imaging. Explicit evaluation across time is critical to ensure model robustness. Nonetheless, several design choices in our study were intended to anticipate potential sources of temporal shifts relevant to clinical deployment. First, the dataset was intentionally heterogeneous, spanning multiple centers and acquisition protocols, incorporating variability in scanner vendors, reconstruction kernels, slice thickness, and contrast use. This diversity partially addresses shifts that may arise over time due to evolving imaging hardware and acquisition practices. Second, established methodological safeguards were implemented in the experimental design, including stratified random splits by center and z-resolution for both the independent train/test partitions and for each k-fold cross-validation split, with strict enforcement of patient-level independence across all sets. Finally, the inclusion of an external cohort provided an additional layer of validation, directly assessing generalizability across centers and acquisition settings distinct from those in the development cohort. We acknowledge, however, the importance of explicit temporal validation strategies, including splits across acquisition years and robustness checks across treatment eras. Hence, these analyses will be a focus of future work to better anticipate model performance under evolving clinical workflows and imaging standards prior to clinical deployment [6].

Finally, the relevance of fairness and subgroup-level robustness is backed by results on both the independent test set and the external cohort provide an initial, robust assessment of the proposed model’s performance. We fully agree that fairness and subgroup-level analyses are important to assess potential disparities, particularly before clinical deployment. As noted in the discussion section and aligned with our adherence to CLAIM reporting elements, we recognize this as a limitation of the current study. Further analyses of fairness and robustness across patient subgroups could represent a valuable direction for future research, especially once more complete clinical metadata is available [7].

We thank Huang and Gu once again for their thoughtful comments, which highlight important avenues for future work toward clinical translation. Overall, we believe our study establishes a solid methodological foundation for multi-instance lung cancer lesion segmentation by integrating lesion- and patient-level analyses, cross-center validation, external cohort evaluation, ablation studies, and comparison against state-of-the-art methods.
